# Sphingosine-1-Phosphate Promotes Extravillous Trophoblast Cell Invasion by Activating MEK/ERK/MMP-2 Signaling Pathways via S1P/S1PR1 Axis Activation

**DOI:** 10.1371/journal.pone.0106725

**Published:** 2014-09-04

**Authors:** Weiwei Yang, Qinghua Li, Zhifang Pan

**Affiliations:** 1 Pharmacy and Biological Science School, Weifang Medical University, Weifang, China; 2 School of Public Health, Weifang Medical University, Weifang, China; Medical University of South Carolina, United States of America

## Abstract

Successful placentation depends on the proper invasion of extravillous trophoblast (EVT) cells into maternal tissues. Previous reports demonstrated that S1P receptors are expressed in the EVT cells and S1P could regulate migration and function of trophoblast cells via S1P receptors. However, little is known about roles of S1P in the invasion of EVT cells. Our study was performed to investigate S1P effect on the invasion of EVT cells. We used the extravillous trophoblast cell line HTR8/SVneo cells to evaluate the effect. In vitro invasion assay was employed to determine the invasion of HTR8/SVneo cells induced by S1P. MMP-2 enzyme activity and relative level in the supernatants of HTR8/SVneo was assessed by gelatin zymography and western blot. Based on the above, siRNA and specific inhibitors were used for the intervention and study of potential signal pathways, and Real-time qPCR and western blot were used to test the mRNA and protein level of potential signal targets. We found that S1P could promote HTR8/SVneo cell invasion and upregulates activity and level of MMP-2. The promotion requires activation of MEK-ERK and is dependent on the axis of S1P/S1PR1. Our investigation of S1P may provide new insights into the molecular mechanisms of EVT invasion.

## Introduction

Invasion of maternal tissues at the maternal-fetal interface by extravillous trophoblast cells (EVT) plays important roles during the normal placentation and successful maintainment of human pregnancy [1], [2]. EVT cells originate from the cytotrophoblast (CTB) cells and then invade into decidual and upper third of myometrium along with remodeling of the associated spiral arteries [3]. The invasive capability of EVT cells is tightly regulated throughout pregnancy by various growth and regulatory factors within the uterine endometrium microenvironment, primarily the decidual [4]. The regulation was performed in the tight spatial and temporal pattern and disruption in this regulation could lead to adverse outcomes [1], [5]. Studies have shown that factors involved in trophoblast invasion regulation are associated with many gestation complications such as early pregnancy loss [6], [7], [8], preeclampsia [9], [10] and fetal growth restriction [11]. Although it plays pivotal roles for successful gestation, the mechanisms underlying the regulation of EVT invasion are not clear, however, it is reported that the invasive capacities of EVT cells are regulated by several factors [12], [13], [14], [15].

Sphingosine-1-phosphate (S1P) is a signaling molecule phosphorylated from spingosine by sphingosine kinases (SPHKs) in most cells [16], [17], and it binds to one of five specific G protein-coupled receptors (S1PR1-5) to activate diverse downstream signaling pathways such as extracellular signal-regulated kinase (ERK), phosphoinositide 3-kinase (PI3K) and phospholipase C (PLC) [18], [19]. Distinct receptor combinations are expressed in different cells and tissues, thus initiating differential activation of distinct signaling pathways and regulation of a broad range of fundamental biological processes including proliferation [20], [21], migration/invasion [22], [23] and apoptosis [24], [25], [26]. S1P has been reported to play roles in migration and invasion in many cancer cell lines. For example, S1P induced cell migration and invasion in OVCAR3 and MCF10A cell lines via S1PR1 or S1PR3 [22], [27], but inhibited migration and invasion in B16 melanoma via S1PR2 receptor [28].

Recent reports lead to the speculation that S1P is involved in reproduction [29] and may regulate invasion of EVT cells. Yamamoto *et al.* reported that there was an increased expression of decidual SPHK1 that could produce S1P in cells and may cause an elevation in deicdual S1P levels in human pregnancy [30]. The results of K. Al-Saghir *et al* and Goyal *et al* demonstrated that there are expressions of S1P receptors (S1PR1-5) in human EVT cells [31], [32], suggesting that S1P may play roles in the regulation of EVT cells. Furthermore, it was reported that migration of EVT cells is inhibited by S1P via S1PR2 [33].

Based on the above evidences, we hypothesized that S1P might regulate EVT invasion. In our study, we focused on the effect of invasion by S1P in human EVT cells. We found that S1P stimulated invasion and MMP-2 expression of HTR8/SVneo cells. Activation of MEK-ERK pathways by S1P is required for S1P-stimulated invasion, and it is dependent of S1P/S1PR1 axis activation.

## Materials and Methods

### Cell Culture and Treatment

The immortalized human EVT cell line, HTR8/SVneo, was a kind gift from Dr. CH Graham at Queen's University, Canada [34]. Cells were cultured in RPMI1640 medium (Invitrogen, Carlsbad, CA) containing 10% fetal bovine serum (FBS), 100 IU/ml penicillin and 100 µg/ml streptomycin, and incubated under 5% CO_2_ at 37 °C. For gelatin zymography assay, cells were cultured in serum-free media. All medium, FBS and enzymes were obtained from Invitrogen unless otherwise noted.

S1P (Sigma-Aldrich, USA) was reconstituted in methanol at 10 µmol/L and stored at −20°C. Cells were trypsinized and then plated in 48-well plates (50,000 cells/well). 24 hours prior to cell stimulation, growth medium was replaced with factors-reduced medium (cell basal medium containing 5% charcoal-stripped FCS). Cells were stimulated with S1P or different inhibitors in serum–starved medium (basal medium with 0.5% charcoal-stripped FCS). Specific siRNAs for MMP-2 and S1PR1 were purchased from Santa Cruz (Sant Cruz, CA, USA) and transfected into the cells using Lipofectamine 2000 reagent according to the manufacturer's instruction (Invitrogen, Carlsbad, CA).

### RNA Extraction and Real-time qPCR

Total RNA was extracted using TRIzol reagent (Invitrogen) and 1 µg of total RNA was reverse-transcribed using reverse transcriptase enzyme (TaKaRa, Dalian, China). Real-time qPCR was conducted using the ABI PRISM 7500 sequence detection system (Applied Biosystems, Carlsbad, CA, USA). The primer sequences were presented in Table 1. Real-time qPCR Reaction conditions were as follows: 5 min at 95°C followed by 40 cycles with 5 s at 95°C and 31 s at 60°C. All reactions were triplicate repeated, and the relative mRNA expression levels for target genes were determined using the 2−ΔΔCT method with normalization by GAPDH [35].

**Table 1:** Primer sequences for Real-time qPCR.

	Forward(5′-3′)	Reverse(5′-3′)
MMP-2	CAGGGAATGAGTACTGGGTCTATT	ACTCCAGTTAAAGGCAGCATCTAC
MMP-9	AATCTCTTCTAGAGACTGGGAAGGAG	AGCTGATTGACTAAAGTAGCTGGA
S1PR1	CGAGAGCACTACGCAGTCAG	GAGAGCCTTCACTGGCTTCA
GAPDH	AACTTTGGCATTGTGGAAGG	GTCTTCTGGGTGGCAGTGAT

### Protein Extraction and Western Blotting Analysis

Whole-cell lysates were prepared as described previously [36]. Protein samples were subjected to 10% SDS-PAGE and transferred to nitrocellulose membrane. The membranes were incubated with 5% defatted milk in PBST for 2 hours, and were then incubated with primary antibodies (according to the manufacture's instruction, typically 1∶1000∼1∶4000 diluted with PBST) overnight at 4°C. Being washed in PBST, the membranes were subsequently incubated with horseradish peroxidase-conjugated secondary antibody (Jackson, PA, USA) for 2 hours. The antibodies used included mouse anti-human S1PR1 (Santa Cruz, USA), rabbit anti-human MMP-2, MMP-9, ERK1/2, phosphorylated-ERK1/2 (Abcam, Cambridge, UK), mouse anti-human MEK1/2 and phosphorylated-MEK1/2 (Cell Signaling Technology, Beverly, MA, USA), and mouse anti-human GAPDH (Ambion, Austin, Texas, USA). Signals were detected using an Enhanced Chemiluminescence Plus kit (Thermo Scientific, Rockford, USA) and visualized after exposure to a Kodak film. The films were scanned and band intensities were analyzed by Image J (NIH, USA).

### In Vitro Invasion Assay

In vitro invasion of HTR8/SVneo cells was measured in Matrigel-coated (Becton Dickinson; Franklin Lakes, NJ) transwell inserts (Costar, Cambridge, MA) containing polycarbonate filters with 8-µm pores as previously described [37]. The inserts were precoated with 50 µg Matrigel matrix in accordance with the manufacturer's recommendations. Briefly, The cells were pretreated with a proliferation inhibitor, mitomycin C (Sigma-Aldrich Corp.), 10 µg/ml, for 2 hours. 1×10^5^ cells per well were plated into the upper chamber in 200 µl RPMI 1640 medium without FBS. 800 µl of medium with 10% FBS was placed into the lower well of the chamber. After 24 hr, the remaining cells on top of the transwell were removed with a cotton swab. The filters with invaded cells attached were washed with PBS, fixed in methanol for 10 min, and stained with hematoxylin. Finally, the number of invaded cells was counted under a light microscope in 15 random-selected non-overlapping fields from each chamber at a magnification of 200×. Average cell numbers in each field were used for statistical analyses. Each experiment was performed in triplicate. All experiments were conducted in triplicate and the invasion index was expressed as the percentage of invaded cell number compared with the corresponding control.

### Gelatin Zymography

Enzyme activities of MMP-2 andMMP-9 in the media were tested using gelatin zymography as previous reported [36]. Briefly, cell culture media were standardized according to the protein contents and subjected to 10% SDS-PAGE containing 1 mg/ml gelatin. After electrophoresis, the gel was washed at room temperature for 1 h in 2.5% triton X-100 (v:v), 50 mM Tris-HCl (pH7.5) to remove SDS, and then incubated at 37°C overnight in a buffer containing 150 mMNaCl, 5 mM CaCl2 and 50 mM Tris-HCl (pH7.6). The gel was subsequently stained with 0.1%(w/v) Coomassie Brilliant Blue R- 250, and destained in 10%(v/v) methanol and 5%(v/v) glacial acetic acid to reveal zones with gelatinase activity. The results were analyzed using Image J version 1.47 software (NIH). All zymography experiments were repeated in triplicate.

### Statistical analyses

All statistical analyses were performed by using SPSS 20 statistical software (SPSS). Results were presented as mean ±SEM from at least three separate repeated experiments; each experiment was conducted in three-parallel replicates, unless otherwise noted. Comparisons were performed with a one-way ANOVA unless otherwise noted, and differences were considered significant at *p*<0.05.

## Results

### S1P stimulates HTR8/SVneo invasion

To determine the effect of S1P on trophoblast cell behaviors, we examined cell proliferation and invasion in HTR8/SVneo cells that were treated with various concentrations of S1P.

**Figure 1:**
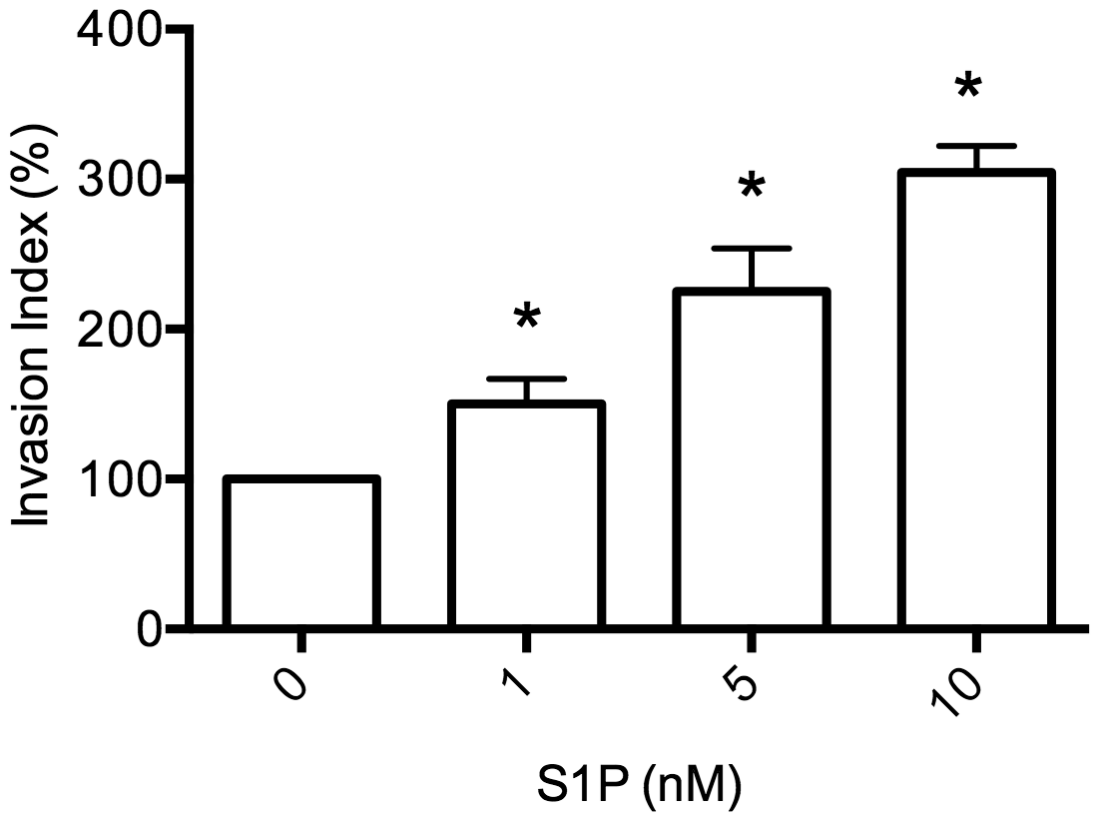
S1P promotes invasion in HTR8/SVneo Cells. The transwell insert invasion assay was performed on HTR8/SVneo cells treated with the indicated concentrations of S1P for 24 hours. Invasion index was expressed as the percentage of invaded cell number compared with the corresponding control. N = 4 performed in triplicate, values were presented as mean ±SEM with *p*<0.05 considered as significant.

Preliminary study found that S1P had no detectable effect on HTR8/SVneo cell viability as measured by MTT assay (data not shown). To get rid of the possibility that the increase in the number of invaded cells under S1P treatment is due to the increase in cell proliferation, mitomycin C was employed to rule out the possible influence of cell growth on the result of cell invasion during transwell insert invasion assay. As shown in Figure 1, invasion was significantly enhanced by S1P treatment in a dose-dependent manner. These results revealed that S1P evidently stimulated cell invasiveness.

### Enhanced invasion of S1P is mediated by MMP-2

**Figure 2:**
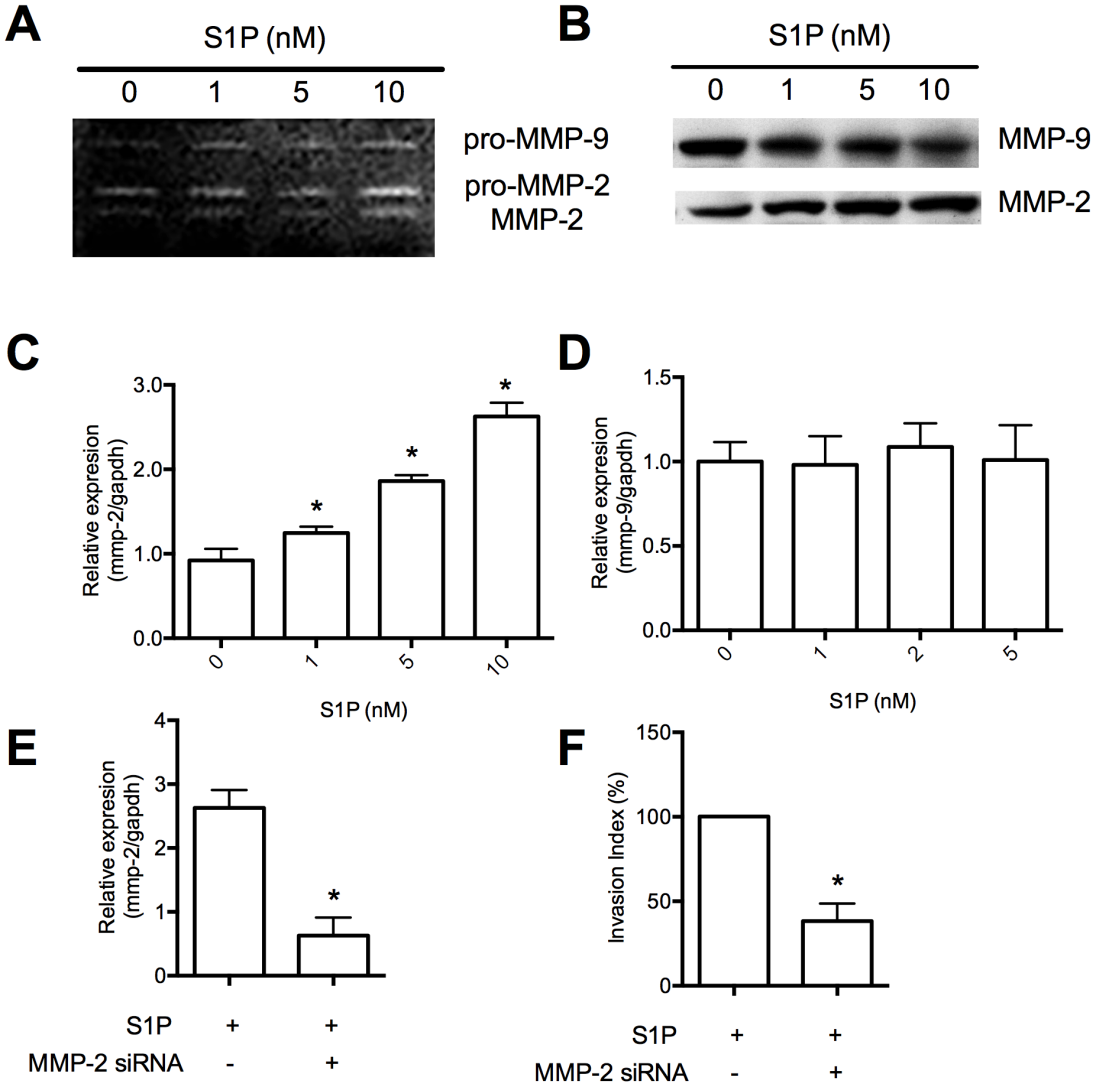
S1P induces MMP-2 expression. Gelatin zymography (A) and western blot (B) that performed on cells treated with S1P for 48 hours demonstrated that MMP-2 expression was induced by S1P. Real-time PCR revealed that expression of MMP-2 (C), but not MMP-9 (D), was induced by S1P treatment for 48 hours in HTR8/SVneo cells. Cells were transfected with control siRNA or siRNA targeting MMP-2. Knockdown expression of MMP-2 was confirmed by Real-time PCR (E). Invasion index was expressed as the percentage of invaded cell number compared with the corresponding control (F). N = 3 performed in triplicate and values were presented as mean ±SEM with *p*<0.05 considered as significant.

Trophoblast cell invasion is often mediated by increased synthesis and activity of MMP-2 and MMP-9; An elevation of MMP-2 was observed in S1P-treated HTR8/SVneo cells using a gelatin zymography (Figure 2A) and western blot (Figure 2B). The mRNA level of MMP-2 was increased by S1P, as evidenced by Real-time PCR, indicating that S1P induces MMP-2 transcriptional expression (Figure 2C). However, we found no detectable effect on MMP-9 expression (Figure 2A, 2B, 2D), suggesting that S1P selectively increases MMP-2, but not MMP-9, in HTR8/SVneo cells.

To further assess the role of MMP-2 in the S1P-induced invasive changes, we knocked-down MMP-2 expression by siRNA technique. Decreased expression of MMP2 was confirmed by gelatin zymography (Figure 2E). Invasion of HTR8/SVneo cells treated with S1P was significantly inhibited by MMP-2 siRNA knock-down (Figure 2F), demonstrating the crucial role of MMP-2 in S1P-induced invasion of HTR8/SVneo cells.

### S1P activates MEK1/2 - ERK1/2 signaling pathways that involves in the invasion of HTR8/SVneo

**Figure 3:**
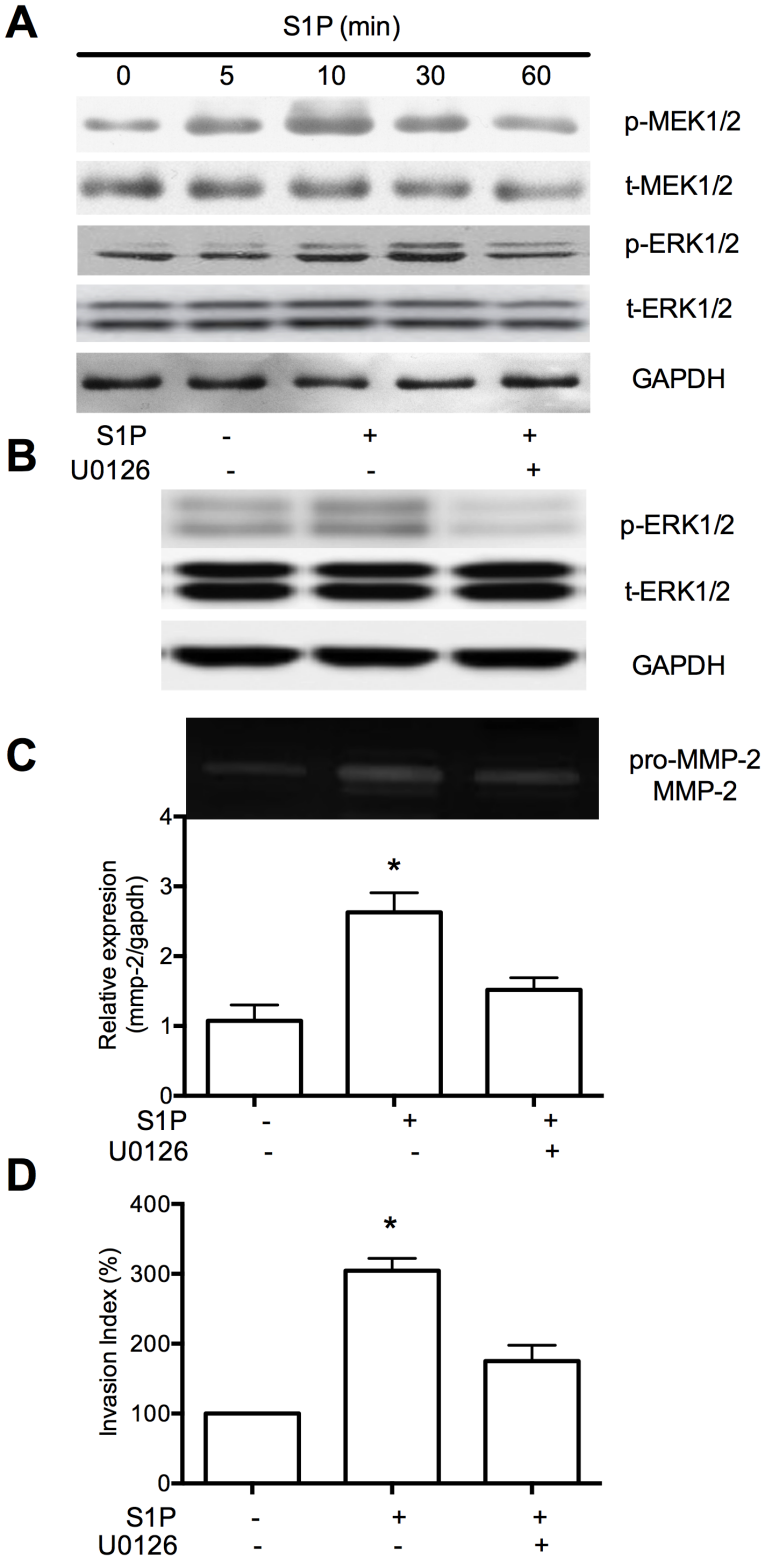
MEK1/2-ERK1/2 signaling pathway is essential for S1P-induced MMP-2 upregulation and invasion of HTR8/SVneo cells. (A) Cells were treated with 10 nM S1P for the indicated time. The level of phosphorylated MEK1/2, ERK1/2 were determined by western blot using phospho-specific antibodies (p-MEK1/2, pERK1/2 respectively). (B), Pretreatment of cells with 10 µM U0126, an inhibitor of MEK1/2, for 60 minutes resulted in significantly less of ERK1/2 phosphorylation (B), which downregulated level of MMP-2 (C). The invasion of MEK1/2 induced by S1P was also inhibited(D). N = 3 performed in triplicate and values were expressed as mean ±SEM with *p*<0.05 considered as significant.

To identify the potential signaling pathways, we investigated the signaling molecules involved in S1P-induced invasion. The levels of the phosphorylated forms of MAPK kinase 1 and 2 (MEK1/2) were markedly increased 10 minutes after S1P treatment and decreased after 30 minutes; The level of the phosphorylated forms of extracellular signal-regulated kinases 1 and 2 (ERK1/2) were increased 10 minutes after S1P treatment and maintained until 30 minutes, then decreased after 60 minutes (Figure 3A). The data indicated that S1P induced activation of MEK1/2, ERK1/2 at different time points in HTR8/SVneo cells.

To address the functional roles of these signaling molecules in S1P-induced MMP-2 upregulation and induction of invasion, Real-time PCR and transwell insert invasion assays were conducted. Treatment with U0126 (an inhibitor of MEK1/2) effectively inhibited the activation of ERK1/2 (Figure 3B). As shown in Figure 3C, zymography and Real-time PCR revealed that U0126 reduced the level of MMP-2. Transwell insert invasion assay indicated that the S1P-induced invasion of HTR8/SVneo cells was also significantly inhibited by U0126 (Figure 3D). These data revealed that ERK1/2 phosphorylation induced by S1P depends on the upstream phosphorylation of MEK1/2; and activation of MEK1/2-ERK1/2 signaling pathways play important roles in S1P-induced upregulation of MMP-2 and invasion of HTR8/SVneo cells.

### Upregulation of MMP-2 and induction of HTR8/SVneo invasion by S1P was mediated by S1PR1

**Figure 4:**
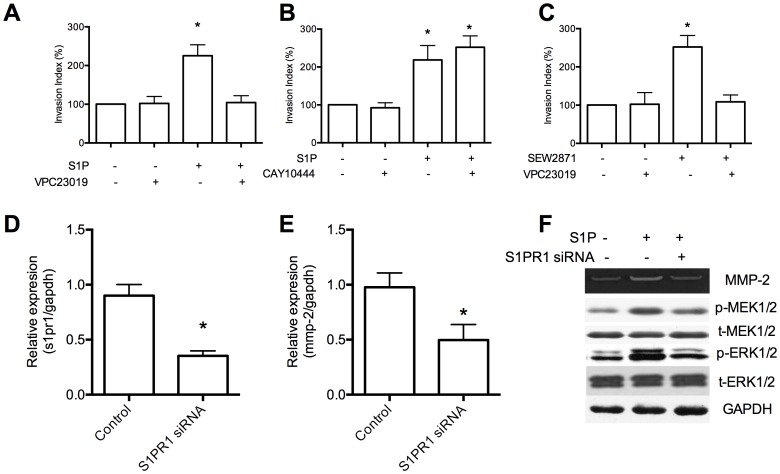
S1P-induced MMP-2 upregulation and signaling pathways is mediated through S1PR1 receptor. (A) HTR8/SVneo cells were pretreated for 30 minutes with the S1PR1 and S1PR3 receptor antagonist VPC23019 (1 µM) before stimulation with S1P (10 nM). (B) HTR8/SVneo were pretreated for 30 minutes with the selective S1PR3 receptor antagonist CAY10444 (1 µM) before stimulation with S1P. (C) HTR8/SVneo were pretreated for 30 minutes with VPC23019 (1 µM) before stimulation with the selective S1PR1 receptor agonist SEW2871 (1 µM). (D) Cells were transfected with control siRNA or siRNAs targeting S1PR1 (50 pmol). Knockdown of S1P1 was confirmed by Real-time PCR. (E) Induction of MMP-2 by S1P was disrupted in siRNA transfected cells. (F) The levels of activated MEK1/2, ERK1/2 were determined by western blot using phospho-specific antibodies (p-MEK1/2, p-ERK1/2, respectively). N = 3 performed in triplicate and values were expressed as mean ±SEM with *p*<0.05 considered as significant.

We tested the effects of S1P receptor specific inhibitor on invasion of HTR8/SVneo cells to identify the involved S1P receptor subtype. The inhibitor of S1PR1 and S1PR3 receptors (VPC23019) inhibited the invasion of HTR8/SVneo induced by S1P (Figure 4A). However, the selective S1PR3 receptor antagonist (CAY10444) did not inhibit S1P-induced invasion (Figure 4B). Furthermore, the selective S1PR1 receptor agonist (SEW2871) stimulated HTR8/SVneo invasion and was inhibited by VPC23019 (Figure 4C). These results suggested that S1P-induced invasion of HTR8/SVneo cells was mediated predominantly via S1PR1 receptor subtype.

To further address the role of S1PR1 in S1P-induced cell response, siRNA targeting S1PR1 gene transcript was employed. The siRNA transfection effectively decreased the level of the S1PR1 mRNA, as confirmed by Real-time PCR (Figure 4D). The S1P-induced upregulation of MMP-2 was significantly inhibited by the siRNA targeting S1PR1 (Figure 4E, 4F). The activation of MEK1/2, ERK1/2 was reduced by knockdown of S1PR1 (Figure 4F). These data demonstrate that S1PR1 is essential for the S1P-induced MMP-2 upregulation and signaling pathways.

## Discussion

Regulation of EVT invasion is pivotal for normal placentation and successful normal gestation. In the present study, we found that S1P stimulated MMP-2 expression and thus promoted invasion of trophoblast cells. Further studies revealed that activation of MEK-ERK signaling pathways is essential for the induction of invasion by S1P, and it is S1P/S1PR1 axis dependent.

Early studies have evidenced that S1P and its signaling pathway were regulated throughout the gestation [38]. Reports have demonstrated that S1P regulates behavior and function in trophoblast cells. Guilbert *et al* reported the first investigation on the effects of S1P on trophoblast function that S1P inhibits placental trophoblast differentiation through G_i_-coupled S1P receptor interactions and reduced production of intracellular cAMP [39]. Ambika *et al* further demonstrated that sphingosine-S1P pathway is involved in the regulation of trophoblast invasion [40]. Goyal suggested that S1P regulates cytokine IL-6 secretion via S1PR2, and further elucidated the underlying mechanism that IL-6 secretion of trophoblast cells induced by S1P is involving Rho and Rac1 signaling pathways [31].

Few evidences have been reported to investigate the effects of S1P on EVT invasion. Previous studies indicated that S1P inhibits EVT migration via S1PR2 [33]. Interestingly, our studies demonstrated that stimulation of S1P results in increased EVT invasion. Besides the distinct inner mechanisms between migration and invasion, the different observations may also be due to different drugs and cell models employed. FTY720 was utilized as the S1P signal treatment factor in Zhang *et al* ‘s studies. FTY720 has been shown to function as an antagonist of S1P receptors, while the phosphorylated form of the compound is a potent agonist [41], [42]. AI-Saghir published their works in the form of meeting abstracts, we had no idea of details. However, AI-saghir reported S1P receptors exression in Swan 71 and SGHPL-4 cell lines in 2010, which thus may be the cell model they employed to investigate migration. Also, as we mentioned previously in the Introduction section, S1P could activates distinct signal pathways via different receptors patterns to induce distinct biological responses.

A key process of cell invasion is the breakdown of ECM by proteases with movement of the invading cell into the cleared space. EVT cells upregulate the expression of proteins that favor uterine wall invasion, including MMPs, VE-cadherin, and HLA-G. The activation of protease activity, especially the gelatinases MMP-2 and MMP-9 is essential to trophoblast invasion and movement through the ECM. Previous reports demonstrated that S1P induced MMP-9 expression in breast cancer cells [27] and MMP-2 expression in endothelial cells [43]. In the present study, we detected no significant altered gelatinases activity and expression of MMP-9. Protein and mRNA levels of MMP-2 produced by HTR8/SVneo cells were increased in response to S1P treatment. The stimulation of S1P to the gelatinases activity of MMP-2 observed with zymography is clearly significant.

In order to fully elucidate the mechanisms involved in S1P-induced EVT invasion, we further investigated the signaling pathways of S1P associated with cellular invasion. Typically, stimulation of S1P could triggers diverse downstream signal pathways [19], [23]. We focus on the ERK, Akt and PLC pathways based on previous studies which shown that these pathways is involved in induced invasion [27], [44]. Our data demonstrated that only phosphorylation of MEK1/2 and ERK1/2 were detected in the induced invasion of HTR8/SVneo cells by S1P. Additionally, pretreatment with U0126, inhibitor of MEK1/2, abrogated the activation of ERK1/2, and thus reverses the S1P-induced MMP-2 expression and invasion of HTR8/SVneo cells. The results suggested that activation of MEK1/2 – ERK1/2 signaling pathway is essential for the induced invasion of S1P in HTR8/SVneo cells.

S1P receptors subtypes differentially regulate cell migratory and invasive response upon drug treatment. S1PR1 and S1PR3 are shown to be involved in migration and invasion of cancer cells [22], [45], [46], [47], whereas S1PR2 plays inhibitory roles in migration and invasion of cancer cell lines [28], [48] and trophoblast cells [33]. Our results demonstrated that upregulation of invasion and MMP-2 expression by S1P is mediated by S1PR1. Given that S1P receptor patterns may be complicated by the presence of multiple isoforms with opposing actions on cell surface, and thus specificity of receptor affinity is a key element in successful S1P receptor-based therapeutic interventions [49], our results implicate the receptor S1PR1 as a potentially valuable therapeutic target for designing new drugs for intervention on invasion of trophoblast.

**Figure 5:**
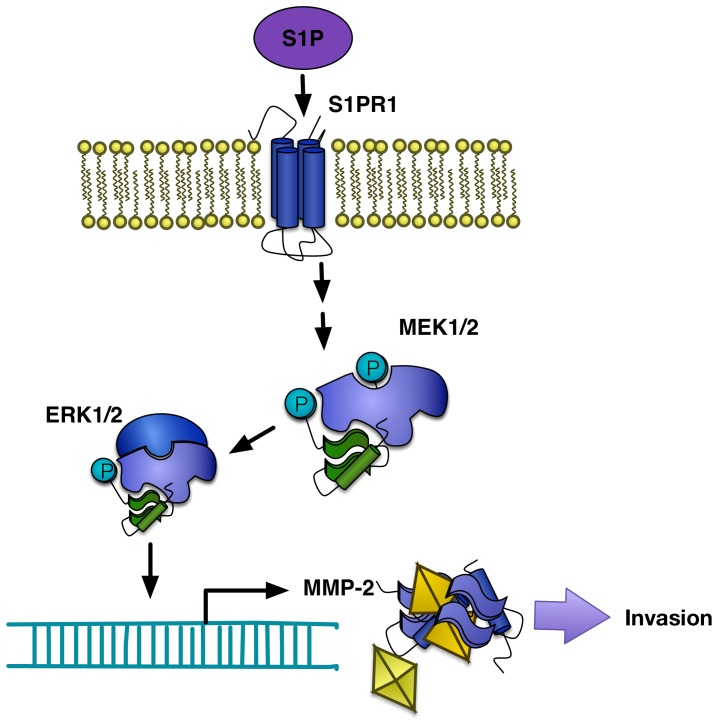
A proposed model of mechanisms involving in S1P-induced invasion of HTR8/SVneo human EVT cells.

On the basis of the observations obtained from this study, we propose a working model of signaling networks that lead to MMP-2 gene expression responsible for the S1P-induced invasion of HTR8/SVneo cells (Figure 5). However, It should be considered when interpreting our results that MMP-2 may not be the sole contributor to the S1P-mediated invasion changes of the cells, because trophoblast invasion involves diverse signaling pathways, such as MMP-9. Recent studies have shown that S1P promotes cell invasion through MMP-9, uPA and MT1-MMP. As we didn't found significant effect on MMP-9 expression in HTR8/SVneo cells, possible contributors would be MT1-MMP and uPA.

Our study was the first to show that S1P promotes invasion of EVT cells via S1PR1 involving activation of MEK-ERK signaling pathway and elevated expression of MMP-2. However, our results must be interpreted under its limitations. We did not exclude the possibility that synthesis of S1P takes place in EVT cells. Many reports indicated that activation of SPHK increases S1P levels, which in turn can function in an autocrine and/or paracrine manners [18]. EVT cells had the potential to synthesis S1P as they show expression of SPHK in trophoblast cells. Given the significant effect of S1P on invasion under relative low doses of S1P, production of local S1P may not be high. In addition, all our experiments were performed *in vitro*, little is known about actions or level of local produced S1P, especially given S1P was produced locally in the decidual tissues [30]. Further study using animal models should be conducted to explore *in vivo* effect of S1P and validate the therapeutic value of potential targets.

In summary, our data demonstrated that S1P could induce EVT invasive phenotypic changes *in vitro*. Induced invasion was correlated with an increase in MMP-2 activity and secretion. Further study characterized the mechanisms by which S1P regulates MMP-2 expression and invasion in the HTR8/SVneo cells. These findings will enhance our understanding of the complex molecular network underlying normal pregnancy and pregnancy-associated disorders.

## References

[1] Anin SA, Vince G, Quenby S (2004) Trophoblast invasion. Hum Fertil (Camb) 7: 169–174. 15590570 10.1080/14647270400006911

[2] Pijnenborg R, Bland JM, Robertson WB, Brosens I (1983) Uteroplacental arterial changes related to interstitial trophoblast migration in early human pregnancy. Placenta 4: 397–413. 6634666 10.1016/s0143-4004(83)80043-5

[3] Pijnenborg R, Dixon G, Robertson WB, Brosens I (1980) Trophoblastic invasion of human decidua from 8 to 18 weeks of pregnancy. Placenta 1: 3–19. 7443635 10.1016/s0143-4004(80)80012-9

[4] Graham CH, Lala PK (1991) Mechanism of control of trophoblast invasion in situ. J Cell Physiol 148: 228–234. 1652588 10.1002/jcp.1041480207

[5] Zhu JY, Pang ZJ, Yu YH (2012) Regulation of trophoblast invasion: the role of matrix metalloproteinases. Rev Obstet Gynecol 5: e137–143. 23483768 PMC3594863

[6] Ball E, Bulmer JN, Ayis S, Lyall F, Robson SC (2006) Late sporadic miscarriage is associated with abnormalities in spiral artery transformation and trophoblast invasion. J Pathol 208: 535–542. 16402350 10.1002/path.1927

[7] Sebire NJ, Fox H, Backos M, Rai R, Paterson C, et al (2002) Defective endovascular trophoblast invasion in primary antiphospholipid antibody syndrome-associated early pregnancy failure. Hum Reprod 17: 1067–1071. 11925407 10.1093/humrep/17.4.1067

[8] Jauniaux E, Burton GJ (2005) Pathophysiology of histological changes in early pregnancy loss. Placenta 26: 114–123. 15708112 10.1016/j.placenta.2004.05.011

[9] Steegers EA, von Dadelszen P, Duvekot JJ, Pijnenborg R (2010) Pre-eclampsia. Lancet 376: 631–644. 20598363 10.1016/S0140-6736(10)60279-6

[10] Crosley EJ, Elliot MG, Christians JK, Crespi BJ (2013) Placental invasion, preeclampsia risk and adaptive molecular evolution at the origin of the great apes: evidence from genome-wide analyses. Placenta 34: 127–132. 23266291 10.1016/j.placenta.2012.12.001

[11] Khong TY, De Wolf F, Robertson WB, Brosens I (1986) Inadequate maternal vascular response to placentation in pregnancies complicated by pre-eclampsia and by small-for-gestational age infants. Br J Obstet Gynaecol 93: 1049–1059. 3790464 10.1111/j.1471-0528.1986.tb07830.x

[12] Tse WK, Whitley GS, Cartwright JE (2002) Transforming growth factor-beta1 regulates hepatocyte growth factor-induced trophoblast motility and invasion. Placenta 23: 699–705. 12398809 10.1016/s0143-4004(02)90866-0

[13] Lash GE, Otun HA, Innes BA, Kirkley M, De Oliveira L, et al (2006) Interferon-gamma inhibits extravillous trophoblast cell invasion by a mechanism that involves both changes in apoptosis and protease levels. FASEB J 20: 2512–2518. 17142800 10.1096/fj.06-6616com

[14] Tapia A, Salamonsen LA, Manuelpillai U, Dimitriadis E (2008) Leukemia inhibitory factor promotes human first trimester extravillous trophoblast adhesion to extracellular matrix and secretion of tissue inhibitor of metalloproteinases-1 and -2. Hum Reprod 23: 1724–1732. 18492704 10.1093/humrep/den121 PMC2474668

[15] Champion H, Innes BA, Robson SC, Lash GE, Bulmer JN (2012) Effects of interleukin-6 on extravillous trophoblast invasion in early human pregnancy. Mol Hum Reprod 18: 391–400. 22362117 10.1093/molehr/gas010

[16] Liu X, Zhang QH, Yi GH (2012) Regulation of metabolism and transport of sphingosine-1-phosphate in mammalian cells. Mol Cell Biochem 363: 21–33. 22113622 10.1007/s11010-011-1154-1

[17] Hannun YA, Obeid LM (2008) Principles of bioactive lipid signalling: lessons from sphingolipids. Nat Rev Mol Cell Biol 9: 139–150. 18216770 10.1038/nrm2329

[18] Maceyka M, Harikumar KB, Milstien S, Spiegel S (2012) Sphingosine-1-phosphate signaling and its role in disease. Trends Cell Biol 22: 50–60. 22001186 10.1016/j.tcb.2011.09.003 PMC3253987

[19] Pyne NJ, Pyne S (2010) Sphingosine 1-phosphate and cancer. Nat Rev Cancer 10: 489–503. 20555359 10.1038/nrc2875

[20] Harada J, Foley M, Moskowitz MA, Waeber C (2004) Sphingosine-1-phosphate induces proliferation and morphological changes of neural progenitor cells. J Neurochem 88: 1026–1039. 14756825 10.1046/j.1471-4159.2003.02219.x

[21] Kim DS, Kim SY, Kleuser B, Schafer-Korting M, Kim KH, et al (2004) Sphingosine-1-phosphate inhibits human keratinocyte proliferation via Akt/protein kinase B inactivation. Cell Signal 16: 89–95. 14607279 10.1016/s0898-6568(03)00114-1

[22] Park KS, Kim MK, Lee HY, Kim SD, Lee SY, et al (2007) S1P stimulates chemotactic migration and invasion in OVCAR3 ovarian cancer cells. Biochem Biophys Res Commun 356: 239–244. 17349972 10.1016/j.bbrc.2007.02.112

[23] Brocklyn JR (2010) Regulation of cancer cell migration and invasion by sphingosine-1-phosphate. World J Biol Chem 1: 307–312. 21537464 10.4331/wjbc.v1.i10.307 PMC3083934

[24] Davaille J, Li L, Mallat A, Lotersztajn S (2002) Sphingosine 1-phosphate triggers both apoptotic and survival signals for human hepatic myofibroblasts. J Biol Chem 277: 37323–37330. 12138095 10.1074/jbc.M202798200

[25] Maceyka M, Payne SG, Milstien S, Spiegel S (2002) Sphingosine kinase, sphingosine-1-phosphate, and apoptosis. Biochim Biophys Acta 1585: 193–201. 12531554 10.1016/s1388-1981(02)00341-4

[26] Cuvillier O, Levade T (2001) Sphingosine 1-phosphate antagonizes apoptosis of human leukemia cells by inhibiting release of cytochrome c and Smac/DIABLO from mitochondria. Blood 98: 2828–2836. 11675357 10.1182/blood.v98.9.2828

[27] Kim ES, Kim JS, Kim SG, Hwang S, Lee CH, et al (2011) Sphingosine 1-phosphate regulates matrix metalloproteinase-9 expression and breast cell invasion through S1P3-Galphaq coupling. J Cell Sci 124: 2220–2230. 21652634 10.1242/jcs.076794

[28] Arikawa K, Takuwa N, Yamaguchi H, Sugimoto N, Kitayama J, et al (2003) Ligand-dependent inhibition of B16 melanoma cell migration and invasion via endogenous S1P2 G protein-coupled receptor. Requirement of inhibition of cellular RAC activity. J Biol Chem 278: 32841–32851. 12810709 10.1074/jbc.M305024200

[29] Guo L, Ou X, Li H, Han Z (2013) Roles of Sphingosine-1-Phosphate in Reproduction. Reprod Sci. 10.1177/1933719113512534 PMC3984488 24336672

[30] Yamamoto Y, Olson DM, van Bennekom M, Brindley DN, Hemmings DG (2010) Increased expression of enzymes for sphingosine 1-phosphate turnover and signaling in human decidua during late pregnancy. Biol Reprod 82: 628–635. 20007411 10.1095/biolreprod.109.081497

[31] Goyal P, Brunnert D, Ehrhardt J, Bredow M, Piccenini S, et al (2013) Cytokine IL-6 secretion by trophoblasts regulated via sphingosine-1-phosphate receptor 2 involving Rho/Rho-kinase and Rac1 signaling pathways. Mol Hum Reprod 19: 528–538. 23538947 10.1093/molehr/gat023

[32] K. Alsaghir DA, M Westwood, E Johnstone (2010) Sphingosine-1-phosphate receptor expression in extravillous trophoblasts. Proc Physiol Soc 19: PC246.

[33] Al-Saghir K, Adlam D, Westwood M, Johnstone E (2013) Sphingosine-1-phosphate (S1P) inhibits extravillous trophoblast migration via S1P receptor 2. Placenta 34: A97–A98. 10.1016/j.placenta.2017.09.009 PMC5754325 29208234

[34] Graham CH, Hawley TS, Hawley RG, MacDougall JR, Kerbel RS, et al (1993) Establishment and characterization of first trimester human trophoblast cells with extended lifespan. Exp Cell Res 206: 204–211. 7684692 10.1006/excr.1993.1139

[35] Livak KJ, Schmittgen TD (2001) Analysis of relative gene expression data using real-time quantitative PCR and the 2(-Delta Delta C(T)) Method. Methods 25: 402–408. 11846609 10.1006/meth.2001.1262

[36] Bai Y, Yang W, Yang HX, Liao Q, Ye G, et al (2012) Downregulated miR-195 detected in preeclamptic placenta affects trophoblast cell invasion via modulating ActRIIA expression. PLoS One 7: e38875. 22723898 10.1371/journal.pone.0038875 PMC3378540

[37] Yang Y, Wang Y, Zeng X, Ma XJ, Zhao Y, et al (2012) Self-control of HGF regulation on human trophoblast cell invasion via enhancing c-Met receptor shedding by ADAM10 and ADAM17. J Clin Endocrinol Metab 97: E1390–1401. 22689693 10.1210/jc.2012-1150

[38] Dunlap KA, Kwak HI, Burghardt RC, Bazer FW, Magness RR, et al (2010) The sphingosine 1-phosphate (S1P) signaling pathway is regulated during pregnancy in sheep. Biol Reprod 82: 876–887. 20107206 10.1095/biolreprod.109.081604 PMC2857631

[39] Johnstone ED, Chan G, Sibley CP, Davidge ST, Lowen B, et al (2005) Sphingosine-1-phosphate inhibition of placental trophoblast differentiation through a G(i)-coupled receptor response. J Lipid Res 46: 1833–1839. 15995175 10.1194/jlr.M500095-JLR200

[40] Singh AT, Dharmarajan A, Aye IL, Keelan JA (2012) Sphingosine-sphingosine-1-phosphate pathway regulates trophoblast differentiation and syncytialization. Reprod Biomed Online 24: 224–234. 22197131 10.1016/j.rbmo.2011.10.012

[41] Brinkmann V, Davis MD, Heise CE, Albert R, Cottens S, et al (2002) The immune modulator FTY720 targets sphingosine 1-phosphate receptors. J Biol Chem 277: 21453–21457. 11967257 10.1074/jbc.C200176200

[42] Paugh SW, Payne SG, Barbour SE, Milstien S, Spiegel S (2003) The immunosuppressant FTY720 is phosphorylated by sphingosine kinase type 2. FEBS Lett 554: 189–193. 14596938 10.1016/s0014-5793(03)01168-2

[43] Sun HY, Wei SP, Xu RC, Xu PX, Zhang WC (2010) Sphingosine-1-phosphate induces human endothelial VEGF and MMP-2 production via transcription factor ZNF580: novel insights into angiogenesis. Biochem Biophys Res Commun 395: 361–366. 20382120 10.1016/j.bbrc.2010.04.019

[44] Bao M, Chen Z, Xu Y, Zhao Y, Zha R, et al (2012) Sphingosine kinase 1 promotes tumour cell migration and invasion via the S1P/EDG1 axis in hepatocellular carcinoma. Liver Int 32: 331–338. 22098666 10.1111/j.1478-3231.2011.02666.x

[45] Young N, Pearl DK, Van BrocklynJR (2009) Sphingosine-1-phosphate regulates glioblastoma cell invasiveness through the urokinase plasminogen activator system and CCN1/Cyr61. Mol Cancer Res 7: 23–32. 19147534 10.1158/1541-7786.MCR-08-0061 PMC2708075

[46] Yamashita H, Kitayama J, Shida D, Yamaguchi H, Mori K, et al (2006) Sphingosine 1-phosphate receptor expression profile in human gastric cancer cells: differential regulation on the migration and proliferation. J Surg Res 130: 80–87. 16183075 10.1016/j.jss.2005.08.004

[47] Paik JH, Chae S, Lee MJ, Thangada S, Hla T (2001) Sphingosine 1-phosphate-induced endothelial cell migration requires the expression of EDG-1 and EDG-3 receptors and Rho-dependent activation of alpha vbeta3- and beta1-containing integrins. J Biol Chem 276: 11830–11837. 11150298 10.1074/jbc.M009422200

[48] Lepley D, Paik JH, Hla T, Ferrer F (2005) The G protein-coupled receptor S1P2 regulates Rho/Rho kinase pathway to inhibit tumor cell migration. Cancer Res 65: 3788–3795. 15867375 10.1158/0008-5472.CAN-04-2311

[49] Kunkel GT, Maceyka M, Milstien S, Spiegel S (2013) Targeting the sphingosine-1-phosphate axis in cancer, inflammation and beyond. Nat Rev Drug Discov 12: 688–702. 23954895 10.1038/nrd4099 PMC3908769

